# Abnormally Low HbA_1c_ Caused by Hemolytic Anemia, a Case Report and Literature Review

**DOI:** 10.3389/bjbs.2024.13898

**Published:** 2025-01-07

**Authors:** Sajjad Bakhtiari, Nathan E. Timbrell, Sènan M. D’Almeida

**Affiliations:** ^1^ Department of Clinical Biochemistry, School of Medicine, Shahid Beheshti University of Medical Sciences, Tehran, Iran; ^2^ Nutristasis Unit, Synnovis, Guy’s and St. Thomas’ NHS Trust, London, United Kingdom; ^3^ Viollier AG, Allschwil, Switzerland

**Keywords:** glycated hemoglobin, HbA_1c_, hemolytic anemia, enzymatic method, diabetes

## Abstract

Hemoglobin A_1c_ is a widely used diagnostic tool for monitoring glycemic control in diabetes management. However, its accuracy can be influenced by various factors. We present a case of a 17-year-old boy with abnormally low Hemoglobin A_1c_ levels caused by warm autoantibody-induced hemolytic anemia. This case highlights the importance of considering conditions that may affect erythrocyte survival, and the potential interferences when interpreting Hemoglobin A_1c_ results to ensure accurate diagnosis and effective management of diabetes.

## Introduction

Hemoglobin (Hb) is the protein contained in red blood cells (RBCs) that is responsible for delivery of oxygen to the tissues. Hb is composed of two pairs of dissimilar chains, α and β, each defined by a specific amino acid sequence and incorporating an iron-containing heme group. Two α–β dimers combine to form a hemoglobin tetramer. In adults, Hemoglobin A (HbA) is the dominant type, accounting for around 97% of all hemoglobin. Minor variations of HbA can arise through post-translational modifications. These modified HbA include A_1a_, A_1b_, and A_1c_, with A_1c_ being the most prevalent of these minor components [1].

Hemoglobin A_1c_ (HbA_1c_) is formed through a non-enzymatic process called glycation, where glucose molecules bind to the amino groups of proteins. Specifically, glucose reacts with the N-terminal amino group of the hemoglobin beta-chain, resulting in the formation of a Schiff base. This reaction then undergoes a rearrangement to form HbA_1c_. Notably, this process is irreversible and depends on both the average glucose levels in the blood and the age of RBCs [2]. RBCs typically have a lifespan of approximately 120 days. Therefore, glycated hemoglobin reflects the average glucose levels over the past 60–90 days [3].

HbA_1c_ concentration is a useful tool for monitoring glycemic control over time, as well as establishing treatment goals and decision boundaries [4, 5]. The HbA_1c_ test indicates the average blood glucose level for the last 8–12 weeks [6]. The American Diabetes Association (ADA) has recommended an HbA_1c_ level of ≥6.5% (47.5 mmol/mol) as the diagnostic threshold for diabetes since 2010 [7]. The recommendation to use HbA_1c_ as a diagnostic test is based on its advantages over traditional glucose tests. HbA_1c_ provides a better overall picture of glycemic exposure and long-term complication risk, and it is less susceptible to variations in biological and preanalytical factors. Moreover, HbA_1c_ levels are not affected by sudden changes in glucose levels caused by acute illnesses or stress, making it a more reliable indicator of glycemic control in these situations [8].

Currently, various HbA_1c_ assay methods are used in clinical practice, and significant efforts have been made towards global standardization. This standardization ensures good performance and reproducibility across different assays. It is achieved through traceability to the National Glycohemoglobin Standardization Program (NGSP) and the reference materials and methods of the International Federation of Clinical Chemistry (IFCC) [9, 10]. Recent guidelines recommend intra-laboratory and inter-laboratory coefficients of variation (CV) of <1.5% and <2.5%, respectively, which are achievable due to the efforts of global standardization programs [3].

Methods for measuring HbA_1c_ can be broadly categorized into three groups based on their assay principles. The first group includes methods that detect the charge differences between glycated and non-glycated hemoglobin, such as ion-exchange high-performance liquid chromatography (HPLC). This method is widely used due to its high precision and ability to provide a complete hemoglobin profile. However, it can be affected by hemoglobin variants, leading to potential inaccuracies in patients with hemoglobinopathies [11]. The second group of methods separates glycated from non-glycated hemoglobin based on structural differences, as seen in affinity chromatography or immunoassays. Affinity chromatography specifically binds glycated hemoglobin, making it less affected by hemoglobin variants compared to ion-exchange HPLC [12]. Immunoassays, which use antibodies to recognize glycated hemoglobin, are both rapid and suitable for high-throughput laboratories. However, these methods can be prone to interference from endogenous antibodies, such as heterophile antibodies, which may result in inaccurate measurements [13, 14]. The third group of methods measures HbA_1c_ based on its chemical reactivity, such as enzymatic assays. These methods offer the advantage of being less affected by hemoglobin variants or other structural alterations, and they tend to be quicker and simpler to perform than chromatographic methods [15].

The accuracy of HbA_1c_ measurements can be affected by various factors. Pre-analytical factors can be classified into four primary categories: 1) erythropoiesis factors, such as folate and vitamin B12 deficiency; 2) hemoglobinopathies; 3) factors influencing glycation, such as alcoholism, renal failure, and the consumption of vitamins C and E; and 4) factors related to erythrocyte destruction, such as hemolytic anemia and certain drugs. Analytical factors include variations in reagent lot or those specific to antibody-based methods such as the presence of heterophile antibodies [9, 16]. Heterophile antibodies are non-specific antibodies that may bind to reagents used in the assay, such as monoclonal or polyclonal antibodies, leading to false results. Depending on the specific assay technique, these antibodies can either enhance or suppress the signal, resulting in inaccurate HbA_1c_ measurements [14].

These factors may influence the measurement of HbA_1c_ based on the method principle and it is essential to consider these factors when interpreting HbA_1c_ results to ensure accurate conclusions. In this study, we present a case of abnormally low HbA_1c_ caused by hemolytic anemia in a 17-year-old boy.

## Case Description

Following a clinical examination that revealed abdominal pain, lateral edema, and splenomegaly, a 17-year-old boy presented to the laboratory for a blood test under a physician’s prescription. The patient denied any significant medical history or recent drug use. He underwent routine laboratory tests, including screening for glucose metabolism, with HbA_1c_ included as part of the standard check-up. During the investigation, an abnormally low HbA_1c_ level, despite a normal fasting plasma glucose (FPG) level, was noted. HbA_1c_ was measured using a modified enzymatic method, showing a significant decrease to 2.8% (7.0 mmol/mol), while the FPG level was 92 mg/dL (reference range: 70–106 mg/dL), which was discordant with the HbA_1c_ result. The patient had no family history of hyper- or hypoglycemia.

Hematological findings revealed normocytic, normochromic anemia, with platelet (PLT) and white blood cell (WBC) counts remaining within the reference range (Table 1). Examination of the peripheral blood smear, stained with new methylene blue, demonstrated significant reticulocytosis at 9.8%.

**TABLE 1:** Hematology findings of the patient.

Test	Result	Reference range
RBC (x10^12^/L)	**2.56**	4.0–5.5
Hb (g/L)	**81**	120–160
HCT (L/L)	**0.25**	0.37–0.49
MCV (fL)	90.8	80–100
MCH (pg)	31.5	27–34
MCHC (g/L)	347	320–360
WBC (x10^9^/L)	6.7	3.5–11.0
PLT (x10^9^/L)	234	180–345
Reticulocyte (%)	**9.8**	0.5–2.5

Values in bold indicate results outside the reference interval.

The biochemical findings revealed mild elevations in serum alanine aminotransferase (ALT), aspartate transaminase (AST), and lactate dehydrogenase (LDH) levels and a significant increase in indirect bilirubin. Other biochemistry tests were in the reference range as shown in Table 2.

**TABLE 2:** Biochemistry findings of the patient.

Test	Result	Reference range
HbA1c (%)	**2.8**	4.0–6.0
FPG (mmol/L)	5.1	3.9–5.9
Urea (mmol/L)	7.1	3.0–7.5
Creatinine (µmol/L)	88.5	61.9–123.9
ALT (U/L)	**62**	<40
AST (U/L)	**78**	<37
LDH (IU/L)	**436**	<280
Total bilirubin (µmol/L)	**116.2**	1.7–20.5
Direct bilirubin (µmol/L)	**10.2**	1.7–5.1
TSH (mIU/L)	2.2	0.5–5.0
T4 (nmol/L)	79.3	60–150
Ferritin (µg/L)	71.7	16–220
25 (OH)D_3_ (nmol/L)	126.3	75–250 (sufficient)

Values in bold indicate results outside the reference interval.

In this patient, the diagnosis of hemolytic anemia was unexpected. The direct antiglobulin test (DAT) revealed the presence of warm autoimmune hemolytic anemia. After exclusion of lymphoproliferative disorders, rheumatic disorders, non-lymphoid malignancies, and drug-induced autoimmune hemolytic anemia, it was classified as idiopathic [17].

## Discussion

**FIGURE 1:**
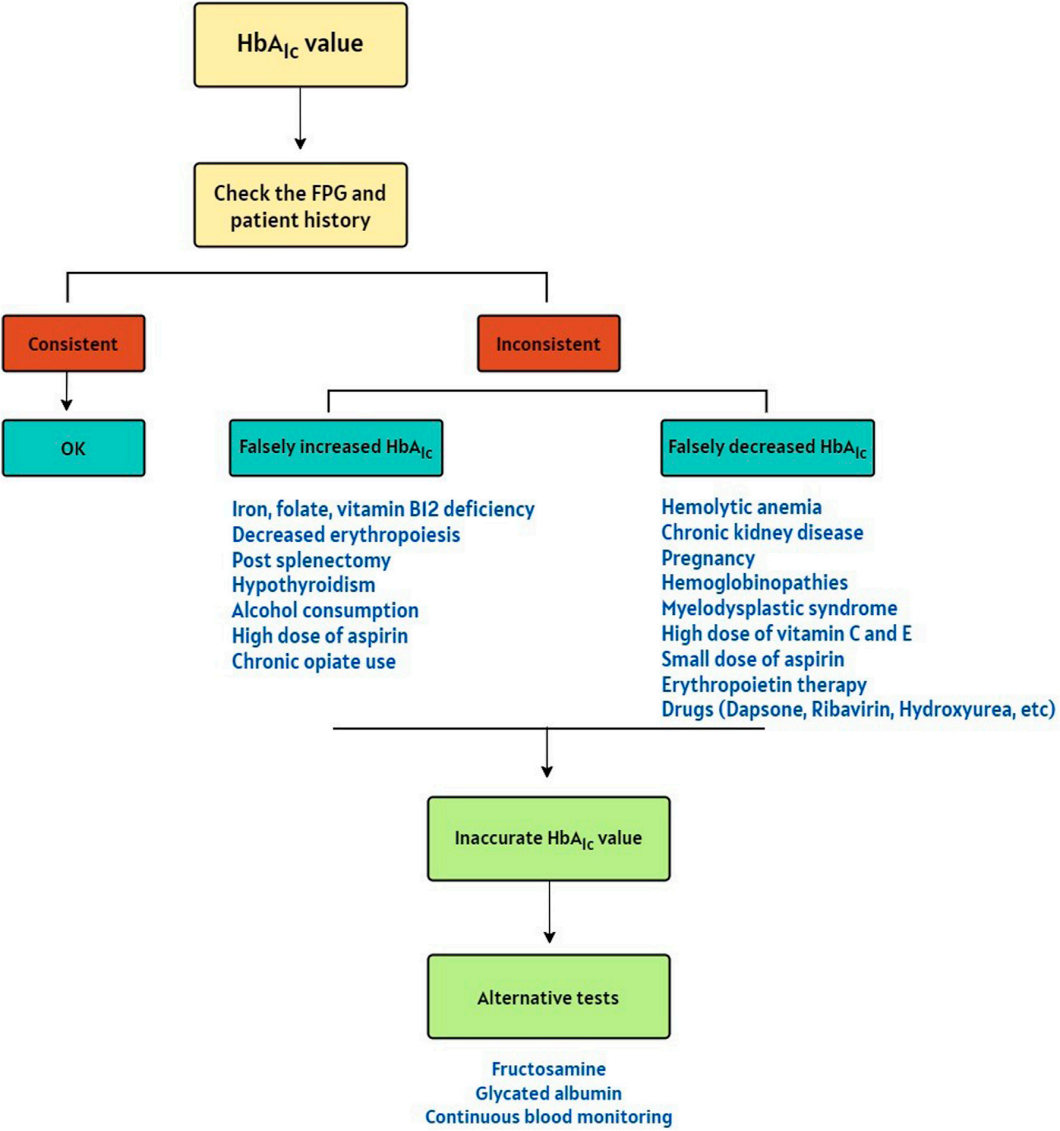
A Decision algorithm for suspicion of the falsely increased or decreased HbA_1c_ results [18–20].

HbA_1C_ is a useful tool for glycemic control in individuals with diabetes mellitus. Discrepancies between HbA_1C_ value, FPG levels, and patient history should prompt consideration of underlying factors that could lead to inaccurate HbA_1c_ results. While HbA_1c_ is generally a reliable indicator of glycemia, there are specific circumstances where its reliability is compromised (Figure 1). Interfering factors are typically method-dependent and may lead to overestimation or underestimation of HbA_1c_ in various mechanisms.

As in this case, hemolytic anemia is a condition marked by the premature destruction of erythrocytes, which can occur either intravascular or extravascular, primarily within the reticuloendothelial system [21]. This normocytic normochromic anemia is usually characterized by elevated indirect bilirubin, elevated LDH, decreased haptoglobin, increased reticulocyte count, and the presence of spherocytes in blood smear [22]. Warm autoantibody-induced hemolytic anemia can affect people of all ages, but this condition is more prevalent among those over 40 years old, with the peak incidence typically occurring in the 70s [23]. However, a study conducted at the Mayo Clinic from 1994 to 2014 on 35 pediatric patients (median age, 10 years old) with autoimmune hemolytic anemia revealed that warm antibodies were the underlying cause of hemolytic anemia in approximately 80% of the patients, consistent with our case [24]. Warm antibody hemolytic anemia is the primary type of autoimmune hemolytic anemia, accounting for approximately 80%–90% of cases. In this condition, warm-reactive antibodies, which are most active at body temperature (37°C), bind to RBCs and initiate a complement-mediated process that damages the cell membranes [25].

Hemoglobinopathies are the leading global cause of inherited single-gene disorders worldwide that are often associated with artificially altered HbA_1c_ levels [26]. If a patient does not have HbA, as is the case in individuals with homozygous variants such as sickle cell disease, HbA_1c_ testing should be avoided, and alternative tests should be used. For other variants, guidelines recommend that laboratories should be aware of the potential effects of hemoglobinopathies on their selected methods [3]. Some methods used to measure HbA_1c_ can produce inaccurate results in patients with hemoglobinopathies, as these disorders can cause variant hemoglobin molecules to migrate similarly to HbA_1c_ during testing, leading to co-elution and falsely increased or decreased results. Hemoglobin variants can also alter the glycation sites and therefore interfere with HbA_1c_ assays [27]. The impact of hemoglobin variants on HbA_1c_ measurements is method-dependent, and since they are present in nearly one-third of patients with diabetes, it is essential to acknowledge their influence [28].

In addition to Hb variants, chemically modified derivatives of Hb can impact the accuracy of these measurements, though the extent of this effect varies widely across different commercially available methods [29]. Carbamylated Hemoglobin (CarbHb) forms through a non-enzymatic reaction and increases with higher levels of urea-derived cyanate. This primarily occurs in patients with chronic kidney disease or those undergoing dialysis. CarbHb can co-elute with HbA_1c_ during assays, potentially leading to falsely elevated results in some methods [30]. The effects of sulfhemoglobin, methemoglobin (MetHb), and acetylated hemoglobin on HbA_1c_ measurements have been studied among chemically modified derivatives. However, these substances interfere less with newer methods. Sulfhemoglobin alters the absorption spectrum, leading to either falsely low or high results in spectrophotometric methods, depending on the assay type. This alteration can occur during the administration of sulfonamides [31]. MetHb, due to its oxidized iron, alters hemoglobin’s optical properties and can interfere with HbA_1c_ assays that use spectrophotometric or colorimetric techniques. Individuals exposed to oxidizing substances or with conditions like methemoglobinemia are more likely to experience interference with their HbA_1c_ measurements from MetHb [32]. Acetylated hemoglobin, formed through reactions with acetyl groups (such as those from acetylated drugs like aspirin) can interfere with certain HbA_1c_ assays by altering the charge of hemoglobin. This change may cause it to be misidentified as glycated hemoglobin, resulting in falsely elevated HbA_1c_ levels [33].

Drugs can affect HbA_1c_ levels in multiple ways such as oxidation of hemoglobin, subclinical hemolysis, shortened survival of erythrocytes, etc. Despite the theoretical possibility, there have been only a few documented instances of drug-induced variability in HbA_1c_ levels reported in the scientific literature, including dapsone, ribavirin, antiretrovirals, aspirin, hydroxyurea, and Trimethoprim-Sulfamethoxazole [18].

Pregnancy, splenectomy, myelodysplastic syndrome, blood loss, folate, and B12 deficiency can affect the accuracy of the HbA_1c_ test by impacting the survival and lifespan of RBCs as another mechanism in which interference can occur. Additionally, circumstances like iron deficiency anemia, and consumption of alcohol, small doses of aspirin, vitamins C and E can influence the glycation process, potentially leading to erroneous HbA_1c_ results [18, 19].

Notably, population data have shown that HbA_1c_ results may be affected by some patient variables. Age-related increases of approximately 0.1% per decade after 30 years of age have been observed in individuals without diabetes [34]. Although research on the impact of ethnicity on HbA_1c_ results is inconsistent, some evidence suggests that Black and Hispanic populations may have higher HbA_1c_ values than White populations at the same level of glycemia [35, 36]. A meta-analysis of 12 studies involving 49,238 individuals has shown that mean HbA_1c_ levels are 0.26%, 0.24%, and 0.08% higher in Black, Asian, and Hispanic individuals, respectively, compared to White individuals [37]. However, another study did not find any difference between Black and White individuals [38]. While seemingly modest (<1%), these differences could have significant clinical implications if these populations’ decision thresholds are not appropriately adjusted. For instance, the observed disparities may lead to an overestimation of HbA_1c_ levels in Black and Asian individuals, potentially resulting in the over-diagnosis of diabetes mellitus. This is particularly critical when HbA_1c_ values are near clinical decision limits, where small differences can influence diagnostic or treatment decisions. Furthermore, elevated HbA_1c_ levels in older individuals without diabetes mellitus may similarly lead to over-diagnosis, potentially exposing individuals to unnecessary treatment and causing increased financial burdens for healthcare systems. Further work is required to elucidate these relationships, but clinical laboratories should be aware of the potential clinical significance of these factors.

The case highlights a key limitation of relying on HbA_1c_ as a standalone diagnostic test for diabetes, as recommended by the ADA and other guidelines. While HbA_1c_’s long-term glycemic representation makes it a valuable diagnostic tool, conditions such as hemolytic anemia that alter red blood cell turnover can lead to misleading results. In this case, the abnormal HbA_1c_ value was identified because it was compared with FPG and clinical findings. Without these additional measures of glycemic control, the low HbA_1c_ value could have been misinterpreted, potentially leading to underdiagnosis or inappropriate clinical decisions. This underscores the importance of integrating HbA_1c_ with other diagnostic approaches. In cases where the HbA_1c_ value does not align with the FPG and patient history, interfering factors may be responsible for over- or under-estimation. These factors are summarized in the decision algorithm depicted in Figure 1. In such situations, alternative methods can be employed to assess glycemic control. Fructosamine and glycated albumin testing measure average blood glucose levels over the past 2-3 weeks, reflecting short-term glucose control more accurately. Self-monitoring of blood glucose also offers a snapshot of blood glucose levels at a specific point in time, allowing patients and healthcare providers to track changes in glucose levels [39]. By combining these methods, healthcare providers can gain a more comprehensive understanding of a patient’s glucose levels and make informed treatment decisions.

This study has limitations that should be acknowledged. While direct insights from the patient regarding their perspective on the condition were not available, it is well-documented that individuals diagnosed with autoimmune hemolytic anemia often experience significant physical and emotional challenges. However, this case report is limited by the lack of follow-up data, including details on therapeutic interventions and patient outcomes. Consequently, we could not evaluate the effectiveness of standard treatments in this specific case. Future studies or reports with comprehensive follow-up are necessary to provide a more holistic understanding of patient management and perspectives in similar contexts.

## Conclusion

In conclusion, HbA_1c_ measurement is a widely used diagnostic tool for monitoring glycemic control in diabetes management. However, it is essential to consider the various influencing and interfering factors that can affect its accuracy. To ensure accurate diagnosis and effective management of diabetes, it is crucial to consider these factors when interpreting HbA_1c_ results. Alternate methods, such as fructosamine, glycated albumin, and glucose monitoring should be used to assess glycemic control when HbA_1c_ results are inaccurate.

### Take-home messages and learning points

• HbA_1c_ is a useful tool for glycemic control
• The ADA has considered HbA_1c_ ≥ 6.5% diagnostic criteria for diabetes
• Hemolytic anemia is normocytic normochromic anemia that results in an inaccurate HbA_1c_ measurement
• Alternative methods in case of inaccurate HbA_1c_ results should be considered

## Data Availability

The original contributions presented in the study are included in the article/supplementary material, further inquiries can be directed to the corresponding author.

## References

[1] Radin MS. Pitfalls in Hemoglobin A1c Measurement: When Results May Be Misleading. J Gen Intern Med (2014) 29:388–94. 10.1007/s11606-013-2595-x 24002631 PMC3912281

[2] Bunn HF Haney DN Kamin S Gabbay K Gallop P. The Biosynthesis of Human Hemoglobin A1c. Slow Glycosylation of Hemoglobin In Vivo. The J Clin Invest (1976) 57(6):1652–9. 10.1172/JCI108436 932199 PMC436825

[3] Sacks DB Arnold M Bakris GL Bruns DE Horvath AR Lernmark Å Guidelines and Recommendations for Laboratory Analysis in the Diagnosis and Management of Diabetes Mellitus. Clin Chem (2023) 69(8):808–68. 10.1093/clinchem/hvad080 37473453 PMC12376302

[4] Gao W Jin Y Wang M Huang Y Tang H. Case Report: Abnormally Low Glycosylated Hemoglobin A1c Caused by Clinically Silent Rare β-Thalassemia in a Tujia Chinese Woman. Front Endocrinol (2022) 13:878680. 10.3389/fendo.2022.878680 PMC9114733 35600576

[5] Weykamp C. HbA1c: A Review of Analytical and Clinical Aspects. Ann Lab Med (2013) 33(6):393–400. 10.3343/alm.2013.33.6.393 24205486 PMC3819436

[6] Little RR Rohlfing C Sacks DB. The National Glycohemoglobin Standardization Program: Over 20 Years of Improving Hemoglobin A1c Measurement. Clin Chem (2019) 65(7):839–48. 10.1373/clinchem.2018.296962 30518660 PMC6693326

[7] Association AD. Diagnosis and Classification of Diabetes Mellitus. Diabetes care (2010) 33(Suppl. ment_1):S62–S9. 10.2337/dc10-s062 20042775 PMC2797383

[8] Committee TIE. International Expert Committee Report on the Role of the A1C Assay in the Diagnosis of Diabetes. Diabetes care (2009) 32(7):1327–34. 10.2337/dc09-9033 19502545 PMC2699715

[9] Little RR Rohlfing CL. The Long and Winding Road to Optimal HbA1c Measurement. Clin Chim Acta (2013) 418:63–71. 10.1016/j.cca.2012.12.026 23318564 PMC4762213

[10] English E Lenters-Westra E. HbA1c Method Performance: The Great Success Story of Global Standardization. Crit Rev Clin Lab Sci (2018) 55(6):408–19. 10.1080/10408363.2018.1480591 30001673

[11] Schnedl WJ Krause R Halwachs-Baumann G Trinker M Lipp RW Krejs G. Evaluation of HbA1c Determination Methods in Patients With Hemoglobinopathies. Diabetes care (2000) 23(3):339–44. 10.2337/diacare.23.3.339 10868862

[12] Stirk H Allen K. Measurement of Glycated Haemoglobin by Boronate-Affinity High-Pressure Liquid Chromatography. Ann Clin Biochem (1999) 36(2):233–4. 10.1177/000456329903600217 10370743

[13] Lakshmy R Gupta R. Measurement of Glycated Hemoglobin A1c From Dried Blood by Turbidimetric Immunoassay. J Diabetes Sci Technol (2009) 3(5):1203–6. 10.1177/193229680900300527 20144437 PMC2769917

[14] Chaabouni K Chaabouni A Marrekchi R Yaich M Naifar M Kacem FH editors. Immunological Interference with Hemoglobin A1c Assay. Lyon, France: Endocrine Abstracts from 21st European Congress of Endocrinology (2019). 10.1530/endoabs.63.EP53

[15] Zechmeister B Erden T Kreutzig B Weber M Joly P Erdmann J Analytical Interference of 33 Different Hemoglobin Variants on HbA1c Measurements Comparing High-Performance Liquid Chromatography With Whole Blood Enzymatic Assay: A Multi-Center Study. Clinica Chim Acta (2022) 531:145–51. 10.1016/j.cca.2022.03.028 35378091

[16] Gallagher EJ Le Roith D Bloomgarden Z. Review of Hemoglobin A1c in the Management of Diabetes. J Diabetes (2009) 1(1):9–17. 10.1111/j.1753-0407.2009.00009.x 20923515

[17] Bass GF Tuscano ET Tuscano JM. Diagnosis and Classification of Autoimmune Hemolytic Anemia. Autoimmun Rev (2014) 13(4-5):560–4. 10.1016/j.autrev.2013.11.010 24418298

[18] Unnikrishnan R Anjana RM Mohan V. Drugs Affecting HbA1c Levels. Indian J Endocrinol Metab (2012) 16(4):528–31. 10.4103/2230-8210.98004 22837911 PMC3401751

[19] Pant V. HbA1c Below the Reportable Range. Lab Med (2022) 53(2):e44–e47. 10.1093/labmed/lmab082 34611711

[20] Campbell L Pepper T Shipman K. HbA1c: A Review of Non-glycaemic Variables. J Clin Pathol (2019) 72(1):12–9. 10.1136/jclinpath-2017-204755 30361394

[21] Dhaliwal G Cornett PA Tierney JLM. Hemolytic Anemia. Am Fam Physician (2004) 69(11):2599–606. 15202694

[22] Tabbara IA. Hemolytic Anemias. Diagnosis and Management. The Med Clin North America (1992) 76(3):649–68. 10.1016/s0025-7125(16)30345-5 1578962

[23] Lum G. Artefactually Low Hemoglobin A1c in a Patient With Hemolytic Anemia. Lab Med (2010) 41(5):267–70. 10.1309/lme5q0lrzdw4dhjr

[24] Sankaran J Rodriguez V Jacob EK Kreuter JD Go RS. Autoimmune Hemolytic Anemia in Children: Mayo Clinic Experience. J Pediatr hematology/oncology (2016) 38(3):e120–4. 10.1097/MPH.0000000000000542 26925716

[25] Packman CH. Hemolytic Anemia Due to Warm Autoantibodies. Blood Rev (2008) 22(1):17–31. 10.1016/j.blre.2007.08.001 17904259

[26] Goonasekera H Paththinige C Dissanayake V. Population Screening for Hemoglobinopathies. Annu Rev genomics Hum Genet (2018) 19(1):355–80. 10.1146/annurev-genom-091416-035451 29751732

[27] Klonoff DC. Hemoglobinopathies and Hemoglobin A1c in Diabetes Mellitus. CA: Los Angeles, CA: SAGE Publications Sage (2020). p. 3–7. 10.1177/1932296819841698 PMC7189151 30897962

[28] Mitchai M Suwansaksri N Seanseeha S Saenboonsiri J Kraitree P Piyapromdee J Misleading HbA1c Measurement in Diabetic Patients With Hemoglobin Variants. Med Sci (2021) 9(2):43. 10.3390/medsci9020043 PMC8293317 34200315

[29] Bry L Chen PC Sacks DB. Effects of Hemoglobin Variants and Chemically Modified Derivatives on Assays for Glycohemoglobin. Clin Chem (2001) 47(2):153–63. 10.1093/clinchem/47.2.153 11159762

[30] Szymezak J Lavalard E Martin M Leroy N Gillery P. Carbamylated Hemoglobin Remains a Critical Issue in HbA1c Measurements. Clin Chem Lab Med (2009) 47(5):612–3. 10.1515/CCLM.2009.136 19397488

[31] Tack CJ Wetzels JF. Decreased HbA1c Levels Due to Sulfonamide-Induced Hemolysis in Two IDDM Patients. Diabetes Care (1996) 19(7):775–6. 10.2337/diacare.19.7.775 8799639

[32] Aljenaee K Hakami O Davenport C Farrell G Tun TK Pazderska A Spurious HbA1c Results in Patients With Diabetes Treated With Dapsone. Endocrinol Diabetes Metab Case Rep (2019) 2019:19-0027. 10.1530/EDM-19-0027 PMC6765317 31566188

[33] Weykamp CW Penders TJ Muskiet FA van der Slik W. Influence of Hemoglobin Variants and Derivatives on Glycohemoglobin Determinations, as Investigated by 102 Laboratories Using 16 Methods. Clin Chem (1993) 39(8):1717–23. 10.1093/clinchem/39.8.1717 7689046

[34] Pani LN Korenda L Meigs JB Driver C Chamany S Fox CS Effect of Aging on A1C Levels in Individuals without Diabetes: Evidence from the Framingham Offspring Study and the National Health and Nutrition Examination Survey 2001-2004. Diabetes Care (2008) 31(10):1991–6. 10.2337/dc08-0577 18628569 PMC2551641

[35] Ziemer DC Kolm P Weintraub WS Vaccarino V Rhee MK Twombly JG Glucose-Independent, Black-White Differences in Hemoglobin A1c Levels: A Cross-Sectional Analysis of 2 Studies. Ann Intern Med (2010) 152(12):770–7. 10.7326/0003-4819-152-12-201006150-00004 20547905

[36] Herman WH Ma Y Uwaifo G Haffner S Kahn SE Horton ES Differences in A1C by Race and Ethnicity Among Patients With Impaired Glucose Tolerance in the Diabetes Prevention Program. Diabetes Care (2007) 30(10):2453–7. 10.2337/dc06-2003 17536077 PMC2373980

[37] Cavagnolli G Pimentel AL Freitas PA Gross JL Camargo JL. Effect of Ethnicity on HbA1c Levels in Individuals without Diabetes: Systematic Review and Meta-Analysis. PLoS One (2017) 12(2):e0171315. 10.1371/journal.pone.0171315 28192447 PMC5305058

[38] Nathan DM Kuenen J Borg R Zheng H Schoenfeld D Heine RJ Translating the A1C Assay into Estimated Average Glucose Values. Diabetes Care (2008) 31(8):1473–8. 10.2337/dc08-0545 18540046 PMC2742903

[39] Speeckaert M Van Biesen W Delanghe J Slingerland R Wiecek A Heaf J Are There Better Alternatives Than Haemoglobin A1c to Estimate Glycaemic Control in the Chronic Kidney Disease Population? Nephrol Dial Transplant (2014) 29(12):2167–77. 10.1093/ndt/gfu006 24470517

[40] Bakhtiari S Timbrell NE Almeida SMD. Abnormally Low HbA1c Caused by Hemolytic Anemia, A Case Report and literature review. Research Square. 10.21203/rs.3.rs-5140298/v1 PMC11745879 39839812

