# Erratum: Abnormally Low HbA_1c_ Caused by Hemolytic Anemia, a Case Report and Literature Review

**DOI:** 10.3389/bjbs.2025.14355

**Published:** 2025-02-06

**Authors:** British Journal of Biomedical Science Production Office

**Affiliations:** Frontiers Media SA, Lausanne, Switzerland

**Keywords:** glycated hemoglobin, HbA_1c_, hemolytic anemia, enzymatic method, diabetes

Due to a production error, an incorrect **Conflict of Interest** Statement was provided. The statement previously read

“Author SD’A was employed by Viollier AG. The remaining authors declare that the research was conducted in the absence of any commercial or financial relationships that could be construed as a potential conflict of interest.”

However, Viollier AG is not a commercial company, therefore the statement should read.

“The authors declare that the research was conducted in the absence of any commercial or financial relationships that could be construed as a potential conflict of interest.”

The publisher apologizes for this mistake. The original version of this article has been updated.

